# Actinobacillus ureae Meningitis in Pediatrics: A Rare Clinical Case

**DOI:** 10.7759/cureus.90417

**Published:** 2025-08-18

**Authors:** Najlae Ouamna, Asmae Lamrani Hanchi, Taoufik Ben Houmich, Mohammed Bouskraoui, Nabila Soraa

**Affiliations:** 1 Microbiology, Mohammed VI University Hospital, Faculty of Medicine and Pharmacy, Marrakech, MAR; 2 Pediatrics, Mohammed VI University Hospital, Faculty of Medicine and Pharmacy, Marrakech, MAR

**Keywords:** actinobacillus ureae, bacterial infection, meningitis, pediatrics, rare case

## Abstract

We report the case of a 12-year-old child with a history of head trauma, followed by two episodes of meningitis caused by *Streptococcus pneumonia*, then *Haemophilus influenza (H. influenzae)*, and two osteoplasties in a neurosurgery department. Currently presenting with the frank acute meningeal syndrome with deterioration of the general condition. A lumbar puncture revealed cloudy cerebrospinal fluid (CSF), pleocytosis, hypoglycorachia, and hyperproteinorachia. A multiplex polymerase chain reaction (PCR) was negative, while culture on enriched media isolated *Actinobacillus ureae (A. ureae)*, identified by mass spectrometry (VITEK® 2 MS, bioMérieux,* *France). The antibiogram showed susceptibility to amoxicillin, ceftriaxone, trimethoprim-sulfamethoxazole, and ciprofloxacin. The patient was treated with ceftriaxone for 10 days, leading to a favorable clinical and biological outcome. The literature reports 27 cases of human infection with *A. ureae*, including 14 cases of meningitis. These cases are often associated with head trauma, neurosurgical procedures, or predisposing factors such as alcoholism and immunosuppression. Diagnosis can be challenging in the absence of pathogen-specific clinical or biological markers; however, advanced techniques such as 16S rRNA sequencing may be helpful when conventional methods fail to identify the pathogen. *A. ureae* remains susceptible to beta-lactam antibiotics, with penicillin and amoxicillin as the treatments of choice. This case emphasizes the importance of considering *A. ureae* meningitis in symptomatic patients with a history of head trauma or neurosurgical interventions, stressing the need for early diagnosis and prompt management to improve patient outcomes.

## Introduction

*Actinobacillus ureae (A. ureae)* is a Gram-negative bacillus first described and isolated from human nasal cultures in 1960 as a variant of *Pasteurella haemolytica*, named *Pasteurella haemolytica var. ureae* [1]. Both *Actinobacillus hominis* (*A. hominis)* and *A. ureae* have been reported as rare commensals of the upper respiratory tract and can occasionally cause serious infections in humans [2,3]. To date, 27 cases of *A. ureae* infections, including 14 cases of meningitis, have been documented in the literature [4]. This report describes a case of *A. ureae* meningitis in a 12-year-old child with a history of cranial trauma and two previous episodes of meningitis.

## Case presentation

A 12-year-old patient with a history of craniofacial trauma in 2022 had no record of post-traumatic pneumococcal vaccination. Three months after the trauma, he developed pneumococcal meningitis, which was treated in a hospital setting. He later experienced a second episode of *Haemophilus influenza* *(H. influenza) *meningitis in December 2023, also requiring hospitalization. The patient underwent frontal osteoplasty in 2023 and a second osteoplasty in 2024 in a neurosurgery department. Additionally, he had a history of undocumented sinusitis in March 2024, for which he received antibiotics.

Symptoms started one day before admission, marked by a sudden onset of fever (39°C), intense headaches unrelieved by analgesics, and vomiting, all in the context of a worsening general condition. Upon admission, the patient was conscious, with a Glasgow Coma Scale (GCS) of 15/15, hemodynamically and respiratory stable (SaO2: 96%, RR: 30 c/min, HR: 110 bpm). No thrush or purpura was noted. The neurological exam showed nuchal rigidity with positive Kernig and Brudzinski signs. There were no sensory or motor deficits. The remaining examinations were unremarkable.

**Figure 1:**
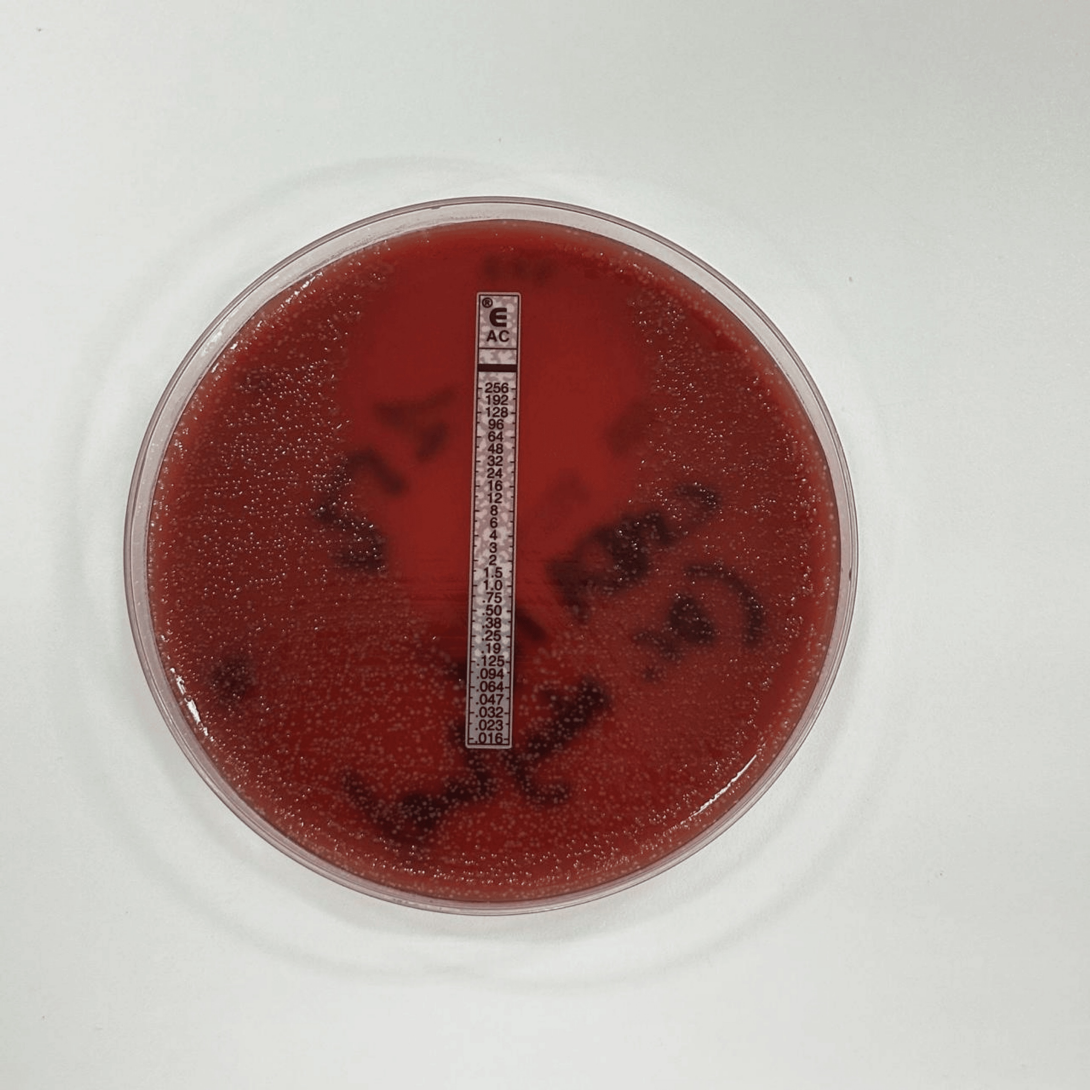
The antibiogram showed susceptibility to amoxicillin, with a minimum inhibitory concentration (MIC) of 0.94 µg/mL.

**Figure 2:**
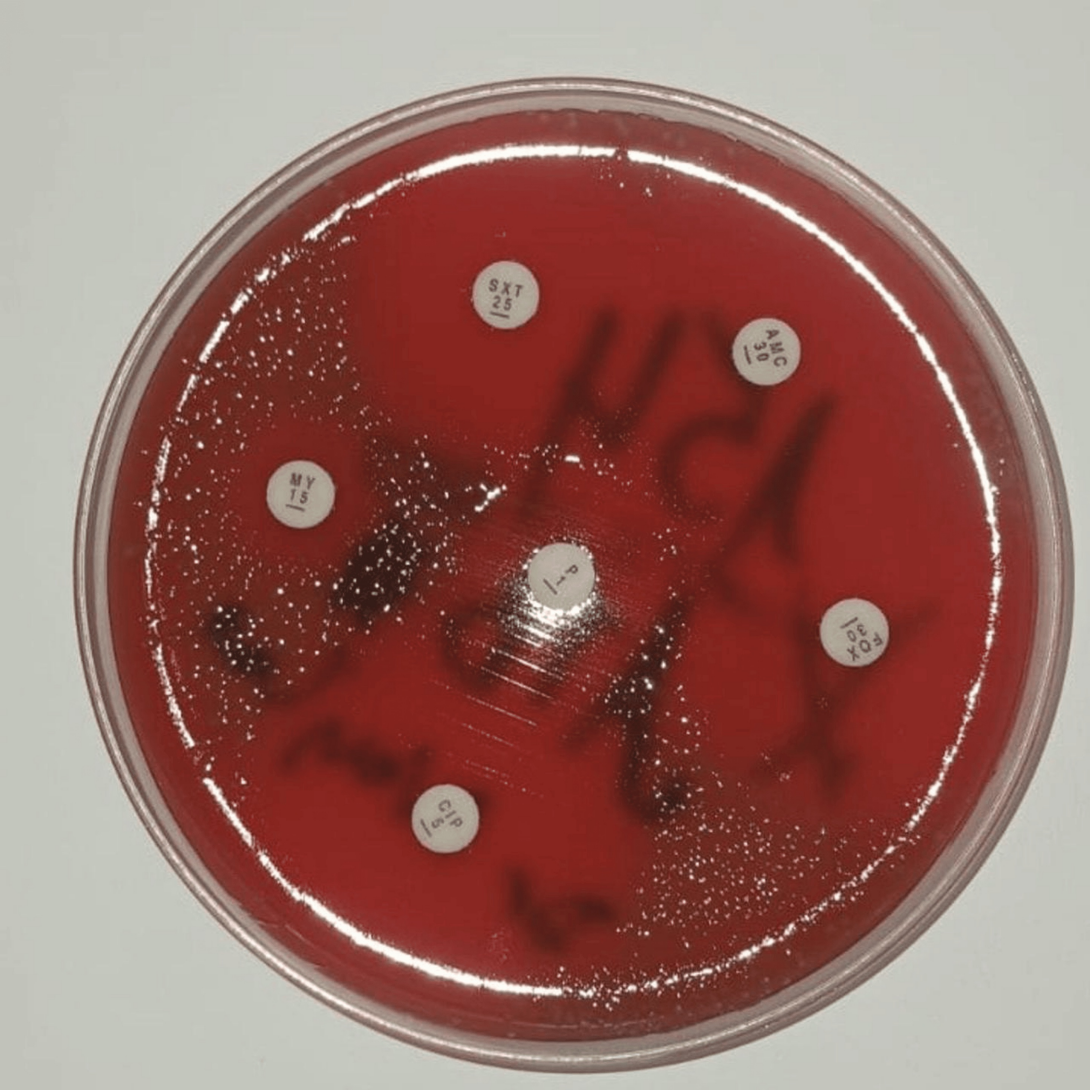
Manual antibiotic susceptibility test.

A brain CT scan performed on admission showed no abnormalities. Complete blood count (CBC) revealed leukocytosis (19,930 cells/mm³) with neutrophil predominance [absolute neutrophil count (ANC): 17,700 cells/mm³]. A lumbar puncture showed cloudy cerebrospinal fluid (CSF) with pleocytosis (6,400 cells/mm³, 80% neutrophils), hypoglycorrhachia (CSF glucose: 0.51 g/L, blood glucose: 1.28 g/L), and hyperproteinorrhachia (2.27 g/L). Direct examination of the CSF did not reveal any microorganisms. A multiplex polymerase chain reaction (PCR) BioFire® FilmArray® meningitis/encephalitis panel was performed on the patient's CSF and was negative. However, culturing the CSF on enriched media led to the isolation of *A. ureae*, identified using mass spectrometry (VITEK® 2 MS, bioMérieux, France). The antibiogram showed susceptibility to amoxicillin, with a minimum inhibitory concentration (MIC) of 0.94 µg/mL, ceftriaxone, trimethoprim-sulfamethoxazole, and ciprofloxacin, but Resistance to lincomycin, as shown in Figures 1, 2, and Table 1.

**Table 1:** Laboratory findings and antibiotic susceptibility. CSF: cerebrospinal fluid, PCR: polymerase chain reaction.

Laboratory Test	Result	Reference Range	Comment
White Blood Cell Count (CBC)	19,930 cells/mm³	4,000–10,000 cells/mm³	Leukocytosis
Absolute Neutrophil Count (ANC)	17,700 cells/mm³	1,500–8,000 cells/mm³	Neutrophilia
CSF Appearance	Cloudy	Clear	Suggestive of infection
CSF White Cell Count	6,400 cells/mm³	< 5 cells/mm³	Marked pleocytosis
CSF Neutrophil Percentage	80%	< 10%	Neutrophilic predominance
CSF Glucose	0.51 g/L	0.5–0.8 g/L	–
Blood Glucose	1.28 g/L	0.7–1.4 g/L	–
CSF/Blood Glucose Ratio	0.40	> 0.6	hypoglycorrhachia
CSF Protein	2.27 g/L	< 0.45 g/L	Elevated (hyperproteinorrhachia)
CSF Culture	Actinobacillus ureae isolated	No growth normally	Identified by mass spectrometry (VITEK® 2 MS)
Multiplex PCR (BioFire® FilmArray®)	Negative	Negative	No pathogens detected
AntibIogram: Amoxicillin	Susceptible (MIC = 0.94 µg/mL)	–	Effective antibiotic
Ceftriaxone	Susceptible	–	Effective antibiotic
Trimethoprim-Sulfamethoxazole	Susceptible	–	Effective antibiotic
Ciprofloxacin	Susceptible	–	Effective antibiotic
Lincomycin	Resistant	–	Ineffective antibiotic

The patient was started on ceftriaxone at a dosage of 100 mg/kg/day in two divided doses for 10 days. This treatment was continued after the identification of *A. ureae* and given its susceptibility to cephalosporins. The clinical outcome was favorable, with a complete resolution of symptoms.

## Discussion

This case adds to the unique yet expanding literature on *A. ureae* meningitis, which remains one of the more overlooked forms of central nervous system (CNS) infection. Only 14 cases have been documented in the literature as of 2024 [5,6]. In most cases of *A. ureae* meningitis, a history of skull fracture, neurosurgical intervention, and chronic alcohol abuse was observed, as well as underlying diseases such as diabetes, hypertension, and schizophrenia. It has been hypothesized that patients with chronic alcoholism, head injuries, and immunosuppression have an increased risk of *A. ureae* infection. Data from the literature indicate that *A. ureae* meningitis should be suspected after head trauma or intracranial surgery, especially in cases of immune dysfunction [7]. This is consistent with our case, in which the patient had a history of craniofacial trauma that occurred three years prior to the current presentation, followed by two neurosurgical interventions. Our report adds to the pediatric population subset, adding the unique consideration of having craniofacial trauma and prior neurosurgical procedures factors which were present in this patient’s history. 

The patient’s history of bacterial meningitis includes infections with *Streptococcus pneumoniae* in 2022 and *H. influenzae* in 2023, coupled with multiple neurosurgical procedures, which likely weakened his protective defenses within the CNS, increasing the risk of more opportunistic pathogens like *A. urea*. Additionally, the presence of sinusitis in early 2024 could have provided yet another route for the pathogen to enter.

There are no specific clinical or laboratory features unique to *A. ureae* meningitis. Direct examination of CSF shows a Gram-negative bacillus, which may be misdiagnosed as *H. influenzae*, which shares several biochemical features with Actinobacillus, and is also a commensal of the upper and lower respiratory tract, and may be a cause of post-traumatic meningitis [8]. Thus, new methods based on 16S rRNA sequencing may be useful in CSF when standard methods fail to identify *A. ureae* [8].

One significant hurdle when diagnosing is that there are no indisputable clinical or laboratory elements that would separate *A. ureae* meningitis from other strains of Gram-negative bacilli. As a matter of fact, the initial mix-up of *H. influenzae* is pretty common due to common overlapping biochemical profiles. This level of diagnostic uncertainty brings to light the need for distinguishing bacterial pathogens with greater speed and accuracy, employing technologies like matrix-assisted laser desorption ionization time-of-flight (MALDI-TOF) mass spectrometry, which was crucial in our case. The failure of the syndromic multiplex PCR panel (BioFire® FilmArray®) to identify the pathogen despite clinical indications of infection highlights a major shortcoming of such targeted diagnostic frameworks: their design to reveal only specific, targeted pathogens does not allow for detection of “unsought” or rare pathogens absent from the included targets. This paradigm, brought forward recently by unconventionally cultured and molecular habitats in 2023, coined the term ‘advanced frameworks’ [9]. Blood cultures were taken at admission and remained negative throughout the entire hospitalization period. Along with the isolation of *A. ureae* from the cerebrospinal fluid (CSF) rather solely reinforces the argument against systemic bacteremia and suggests a more confined central nervous system infection. To rule out contamination, strict aseptic precautions were adhered to during lumbar puncture as well as during the handling of the CSF sample. The organism was identified by culturing on enriched media, and through MALDI-TOF mass spectrometry (VITEK® 2 MS), reliable identification was achieved with reproducible results. The clinical presentation of bacterial meningitis, coupled with the lack of other commensal flora, supports the validity of the finding. 

Notably, MALDI-TOF has increasingly demonstrated clinical utility in infectious disease settings, with studies reporting modifications in empirical therapy in over one-third of bloodstream infection cases based on early identification via this method [10]. Although used here outside of a bloodstream context, this case highlights its expanding role in rapid diagnostics and management across varied infection types.

While utilizing BioFire® FilmArray® meningitis/encephalitis PCR panels, one is confronted with limitations. The panel yielded negative results, which further emphasizes one of the defining limitations of syndromic PCR testing-the inability to identify pathogens outside those specified targets of the test. *A. ureae* is absent from the panel, thus rendering detection impossible through this method. This reinforces the necessity for integrating molecular and conventional culture techniques to achieve thorough investigation in atypical cases.

Based on all previous clinical reports, there are no specific treatment recommendations. *A. ureae* currently remains susceptibility to all β-lactams, and penicillin or amoxicillin appears to be the best treatment. Tetracyclines, macrolides, trimethoprim-sulfamethoxazole, and fluoroquinolones are also active in vitro and could be an alternative in patients allergic to β-lactams. Treatment is based on third-generation cephalosporins (cefotaxime, ceftriaxone) with the possibility of combining with an aminoglycoside depending on the severity of the condition. Microbiological and clinical monitoring is necessary to adapt antibiotic therapy according to the antibiogram. Although other Actinobacillus species, such as Actinobacillus actinomycetemcomitans, can produce β-lactamase, β-lactamase production has not been reported with *A. ureae* [4].

Considering the patient's history of pneumococcal and Haemophilus influenzae meningitis, coupled with the lack of evidence for post-traumatic immunization, catch-up vaccination was strongly suggested.

## Conclusions

This case illustrates the growing significance of *A. ureae* as an infrequent yet notable cause of bacterial meningitis, especially in children with a history of trauma, neurosurgery, or recurrent central nervous system infections. Our results highlight the diagnostic shortcomings of the current multiplex PCR panels due to the infecting organism not being detected, as well as demonstrate the advantage of mass spectrometry-based bacterial identification for atypical, rare, or unforeseen CNS infections. This report, in addition, reinforces the previously documented susceptibility of *A. ureae* to β-lactam antibiotics, while also advocating for more tailored therapy based on culture and susceptibility testing. More research is needed regarding the notion of host factors, such as disrupted anatomical boundaries or immune dampening, in the development of *A. ureae* meningitis. This case further illustrates the importance for clinicians to maintain a high index of suspicion for uncommon pathogens in post-traumatic or post-neurosurgical meningitis while also encouraging the use of alternative diagnostic methods when standard approaches fail, by adding to the modest yet growing collection of literature on this topic.
